# 

**Published:** 1896-06

**Authors:** 


### BOOKS RECEIVED.

Twentieth Century Practice. An International Encyclopedia of Modern Medical Science. By Leading Authorities of Europe and America. Edited by Thomas L. Stedman, M. D., New York City. In twenty volumes. Volume V. Diseases of the Skin. New York: William Wood & Co. 1896.

Weekly Abstract of Sanitary Reports, issued by the Supervising Surgeon-General Marine Hospital Service. Volume X. 1895. Washington: Government Printing Office. 1896.

Transactions of the American Dermatological Association, at its Nineteenth Annual Meeting, held at Montreal, Can., September 17, 18, 19, 1895. New York: Geo. L. Goodman. 1896.

Obstetric Accidents, Emergencies and Operations. By L. Ch. Boisliniere, A. M., M. D., LL.D., late Emeritus Professor of Obstetrics in the St. Louis Medical College; Honorary Fellow of the American Association of Obstetricians and Gynecologists, etc., etc. Duodecimo, pp. 381. Liberally illustrated. Price, $2.00 net. Philadelphia: W. B. Saunders, 925 Walnut street. 1896.

Proceedings of the Philadelphia County Medical Society. Alfred Stengel, M. D., Editor. Volume XVI. Session of 1895. Philadelphia: William J. Dornan, Printer. 1895.

